# Supplementary material for: Charcot–Marie–Tooth type 2A variants of mitofusin 2 sensitize cells to apoptotic cell death
Source: J Cell Sci. 2025 Sep 25;138(18):jcs263691. doi: 10.1242/jcs.263691 (PMC12516130; doi:10.1242/jcs.263691)
Supplement: Supplementary information [file joces-138-263691-s1.pdf]

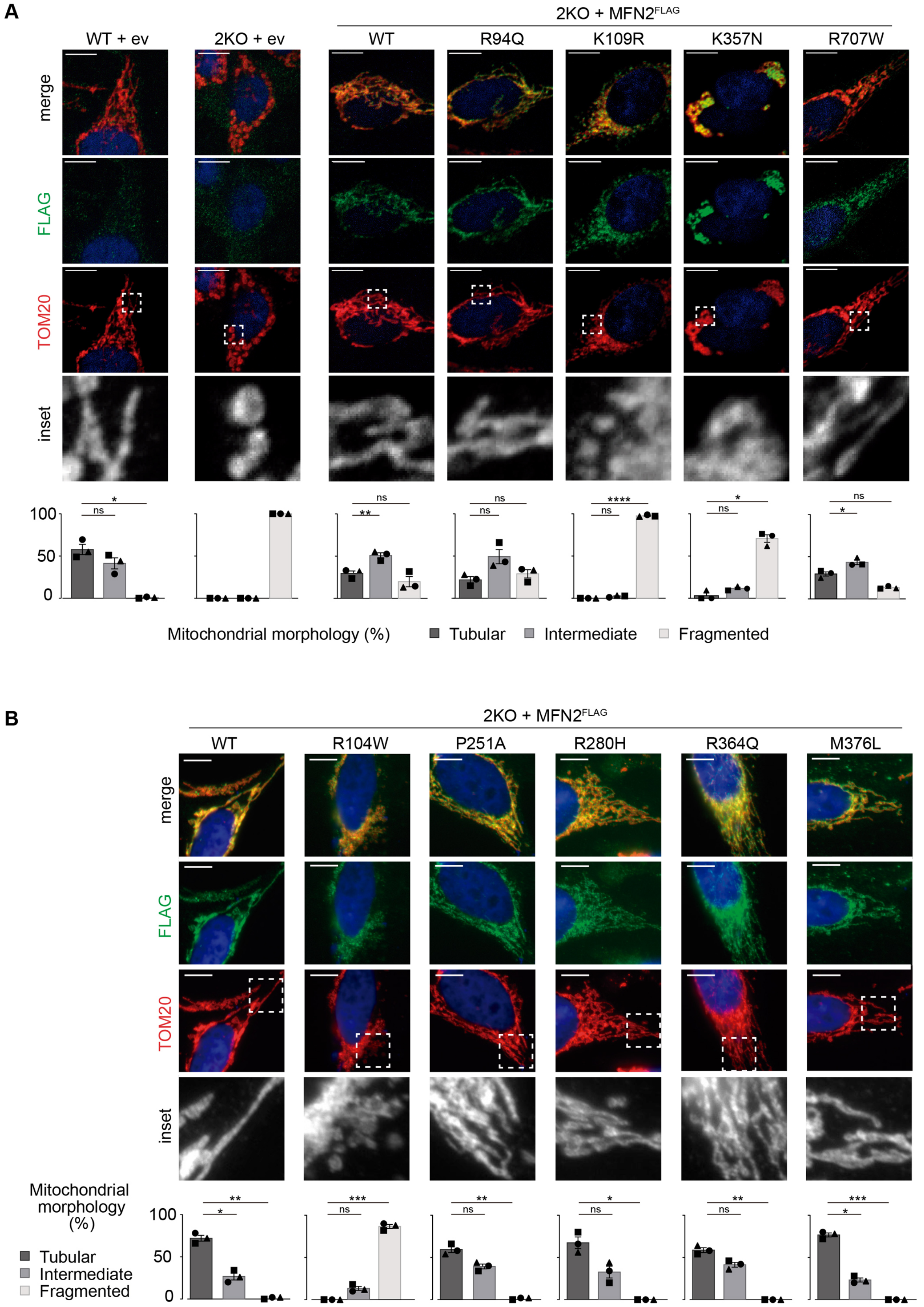

Journal of Cell Science • Supplementary information

Fig. S1. Mitochondrial tubulation is not always affected by CMT2A mutations in MFN2. Confocal images after immunostaining with the outer mitochondrial membrane protein TOM20 (in red), FLAG (in green) and DAPI (in blue), of HeLa WT cells transiently transfected with empty vector (e.v) and 2KO cells transiently transfected with empty vector (e.v) or with FLAG-tagged WT or mutant variants of MFN2: R94Q, K109R, K357N and R707W in **(A)** and R104W, P251A, R280H, R364Q and M376L in **(B)**. Insets of dotted white boxes are shown below the respective images. Mitochondrial morphology was categorized as tubular, intermediate and fragmented. The number of cells showing the respective mitochondrial phenotypes are shown as a percentage of total number of cells. At least 50 cells were counted for quantification of mitochondrial morphology. Individual values of each experiment are discriminated in black filled triangles, circles, or squares. The bars represent the mean percentage of cells with each mitochondrial morphology ± SD (n=3). Scale bar: 10 µm. RM one-way ANOVA was applied. P-values from (A) from left to right: ns 0.5176, * 0.0175, ** 0.0048, ns 0.6086, ns 0.2610, ns 0.1655, ns 0.1177, **** <0.0001, ns 0.2025, * 0.0437, * 0.0905, ns 0.0228. P-values from (B) from left to right: * 0.0478, ** 0.0024, ns 0.0560, *** 0.0003, ** 0.1587, ns 0.0093, ns 0.2273, * 0.0191, ns 0.1702, ** 0.0042, * 0.0146, *** 0.0007.

Journal of Cell Science • Supplementary information

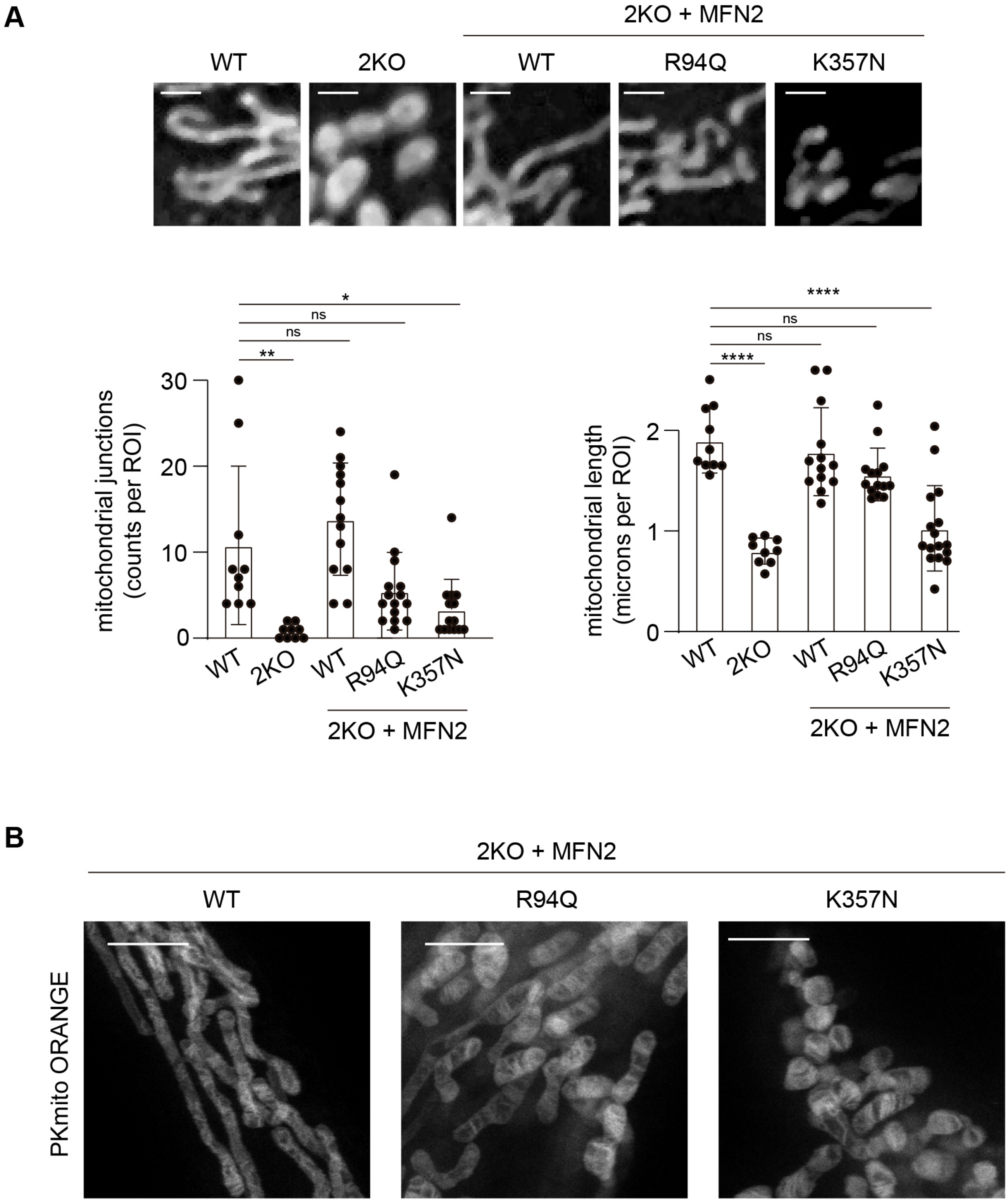
A
2KO + MFN2
WT
2KO
WT
R94Q
K357N
*
ns
ns
**
mitochondrial junctions
(counts per ROI)
30
20
10
0
WT
2KO
WT
R94Q
K357N
2KO + MFN2
****
ns
ns
****
mitochondrial length
(microns per ROI)
2
1
0
WT
2KO
WT
R94Q
K357N
2KO + MFN2
B
2KO + MFN2
WT
R94Q
K357N
PKmito ORANGE

Fig. S2. PKmito ORANGE staining and automated quantification of mitochondrial morphology in CMT2A mutants. (A) Confocal images after live staining with Mitotracker Deep Red of HeLa WT, 2KO and 2KO cells stably expressing FLAG-tagged WT or mutant variants of MFN2 (R94Q or K357N). Examples of regions of interest (ROIs) of each cell line used for mitochondrial morphology assessment are shown. Scale bar: 2μm. Mitochondrial morphology was analysed using the ImageJ/Fiji macro MitoMAPR and mitochondrial length (microns per ROI) and number of mitochondrial junctions (counts per ROI) were quantified (Tabara et al., 2024). At least 10 ROIs were used. Each ROI is discriminated in black filled circles. The bars represent the mean ± SD. Ordinary one-way ANOVA was applied. P values from mitochondrial length quantification from left to right: **** <0.0001, ns 0.9398, ns 0.1499, **** <0.0001. P values from number of mitochondrial junctions quantification from left to right: ** 0.0021, ns 0.6895, ns 0.0166, * 0.1437. **(B)** Confocal images after live staining with PKmito ORANGE of HeLa 2KO cells stably expressing FLAG-tagged WT or mutant variants of MFN2 (R94Q or K357N).
Scale bar: 2μm.

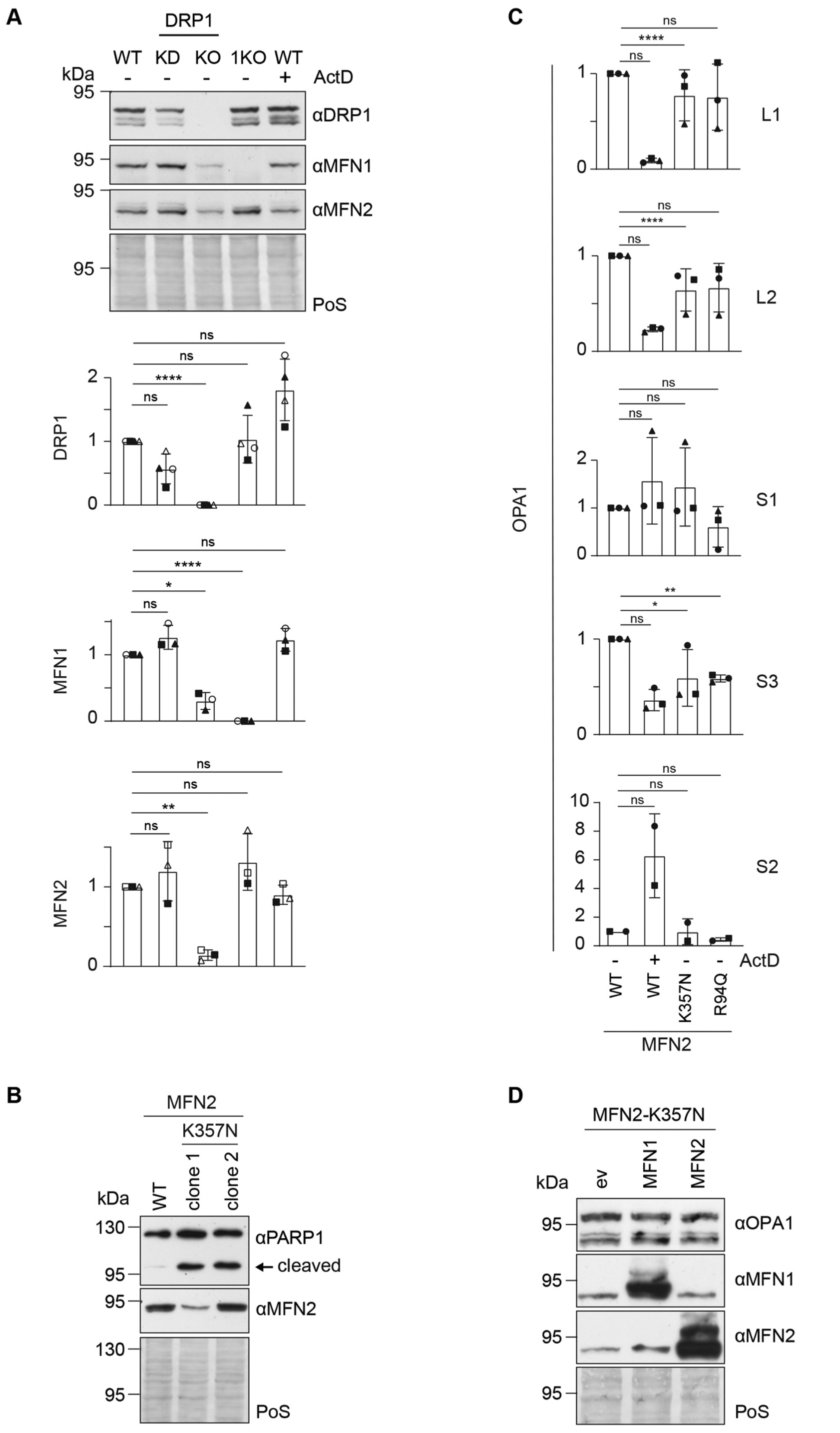

Fig. S3. Interdependence between fusion and fission protein levels. (A) Western blot analysis (upper panel) and quantifications (lower panel) of HeLa WT untreated or treated with ActD (1 µM, 6 h), DRP1 knock-down (KD), DRP1 knock-out (KO) and MFN1 KO (1KO), immunoblotted with anti-DRP1, anti-MFN1 and anti-MFN2. Staining of total protein with PoS was used as loading control. Bars represent the mean intensity, normalized to the PoS, and relative to WT ± SD (n=3-4 biological replicates). Individual values of each experiment are discriminated in empty and filled circles and squares. One-way ANOVA was applied. P values from DRP1 left to right: ns 0.1264, **** <0.0001, ns 0.9995, ns 0.1583. P values from MFN1 from left to right: ns 0.3483, * 0.0331, **** <0.0001, ns 0.3986. P values from MFN2 from left to right: ns 0.8726, ** 0.0065, ns 0.6348, ns 0.6811. **(B)** Western blot analysis of HeLa 2KO cells stably expressing FLAG-

Journal of Cell Science • Supplementary information

tagged WT or the K357N variant (clone 1 and clone 2), immunoblotted with anti-PARP1 and anti-MFN2. Staining of total protein with PoS was used as loading control. **(C)** Quantification of OPA1 long (L1 and L2) and short (S1, S2 and S3) isoforms from western blot analysis presented in Fig. 3B. Individual values of each experiment are discriminated in empty and filled circles and squares. One-way ANOVA was applied. P values for L1 from left to right: **** <0.0001, ns 0.5753, ns 0.6702. P values for L2 from left to right: **** <0.0001, ns 0.2517, ns 0.3441. P values for S1 from left to right: ns 0.7293, ns 0.7973, ns 0.5232. P values for S2 from left to right: * 0.0255, ns 0.3204, ** 0.0070. **(D)** Western blot analysis of HeLa 2KO cells stably expressing K357N mutant variant of MFN2, transiently transfected with empty vector (ev), MFN1 or MFN2, immunoblotted with anti-MFN1, anti-MFN2 and anti-OPA1. Staining of total protein with PoS was used as a loading control.

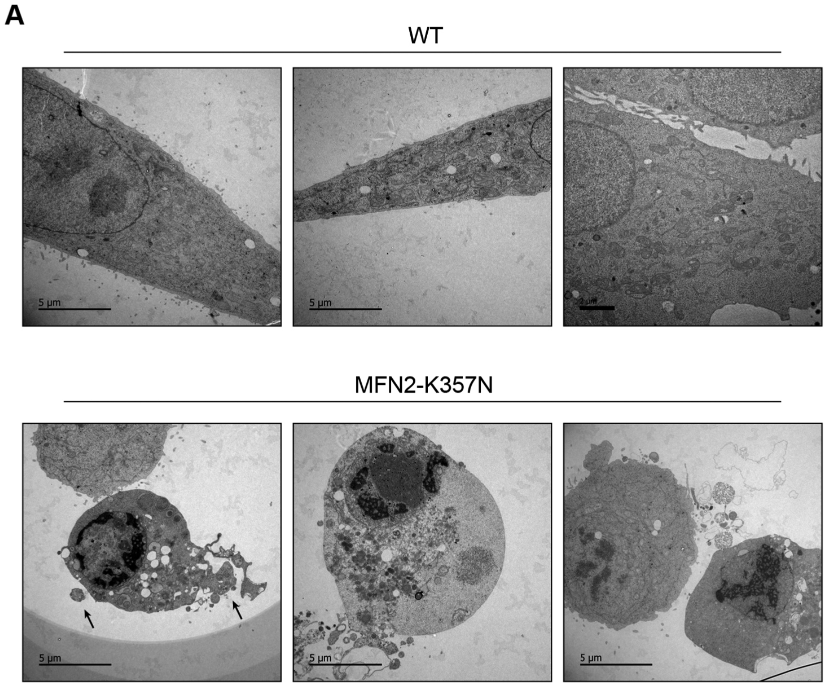

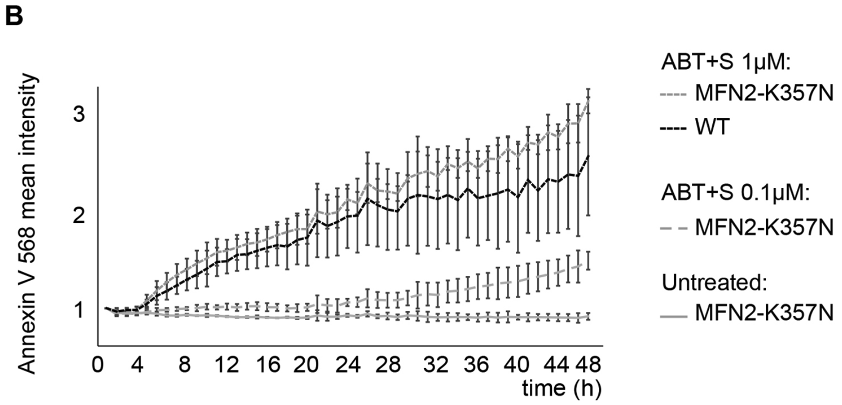

Fig. S4. Apoptotic cell death induction in MFN2-K357N cells. **(A)** Electron microscopy images of HeLa WT and K357N cells. Black and white arrows indicate membrane blebbing and chromatin condensation, respectively. Two magnifications are presented, with scale bars of 5 or 2 µm. **(B)** Annexin V 568 (red) fluorescence, measured by the Incucyte live imaging system over 48 h and relative to timepoint zero, of HeLa WT or 2KO cells stably expressing FLAG-tagged K357N mutant variant of MFN2, untreated or treated with ABT+S (0,1 µM or 1 µM) for apoptosis induction, as indicated. Values represent the mean intensity per object count of 3 technical replicates each from 3 biological replicates.

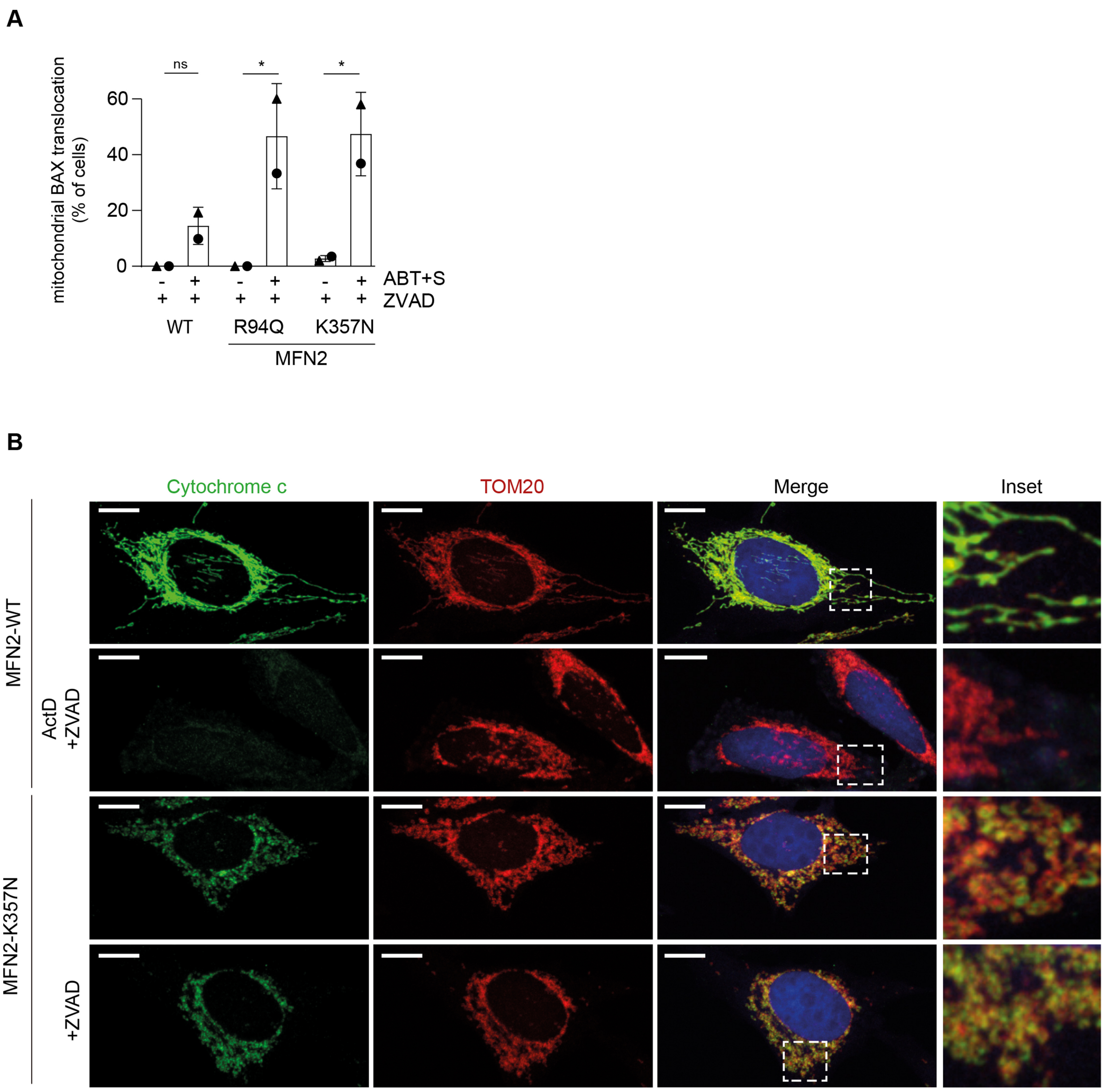

Fig. S5. Immunostaining analysis of apoptosis markers in CMT2A mutant cells. (A) Quantification of mitochondrial BAX translocation (presented in percentage of cells) in HeLa WT and 2KO stably expressing FLAG-tagged mutant variants of MFN2 (R94Q and K357N). At least 50 cells from n=2 biological replicates were used for quantification. Individual values of each experiment are discriminated in black filled triangles and circles. The bars represent the mean percentage of cells with mitochondrial BAX translocation ± SD (n=3). P values from left to right: **(B)** Confocal images after immunostaining with anti-cytochrome C, (in green), TOM20 (in red) and DAPI (in blue), of HeLa WT cells and 2KO cells stably expressing FLAG-tagged K357N mutant variant of MFN2, untreated or treated with ActD (1 μM, 6 h) and/or ZVAD (20 μM, 6 h), when indicated. Insets of dotted white boxes are shown on the right side of the respective images. Scale bar: 10 μm.

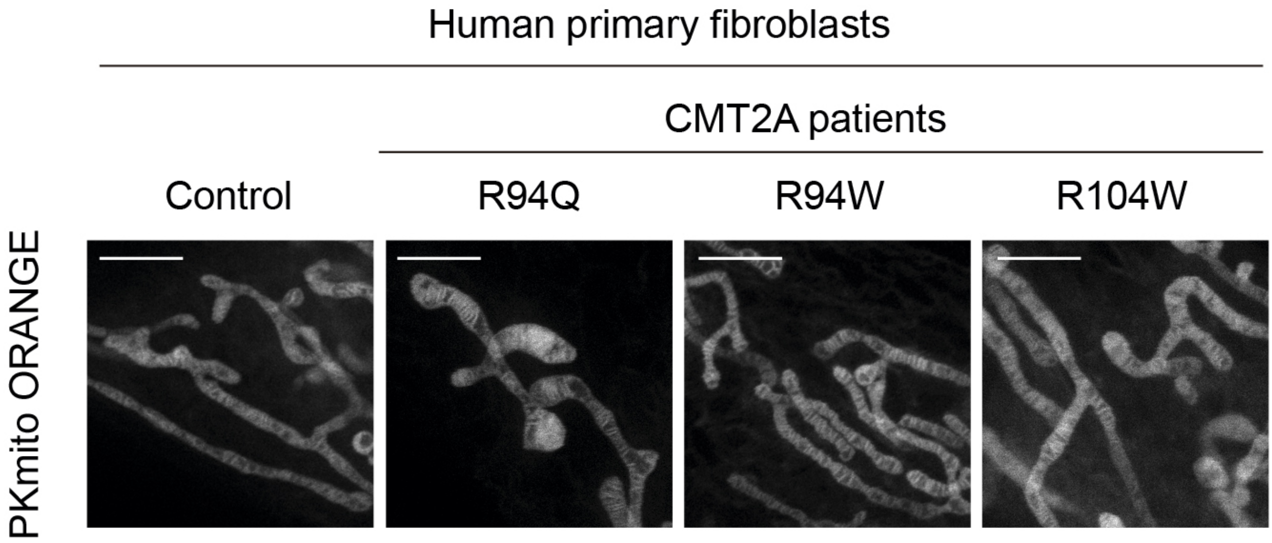

Fig. S6. Mitochondrial morphology of human primary fibroblasts, assessed with PKmito ORANGE staining. Confocal images after live staining with PKmito ORANGE of control or CMT2A patients' primary fibroblasts carrying the point mutations R94Q, R94W or R104W in MFN2. Scale bar: 2 µm.

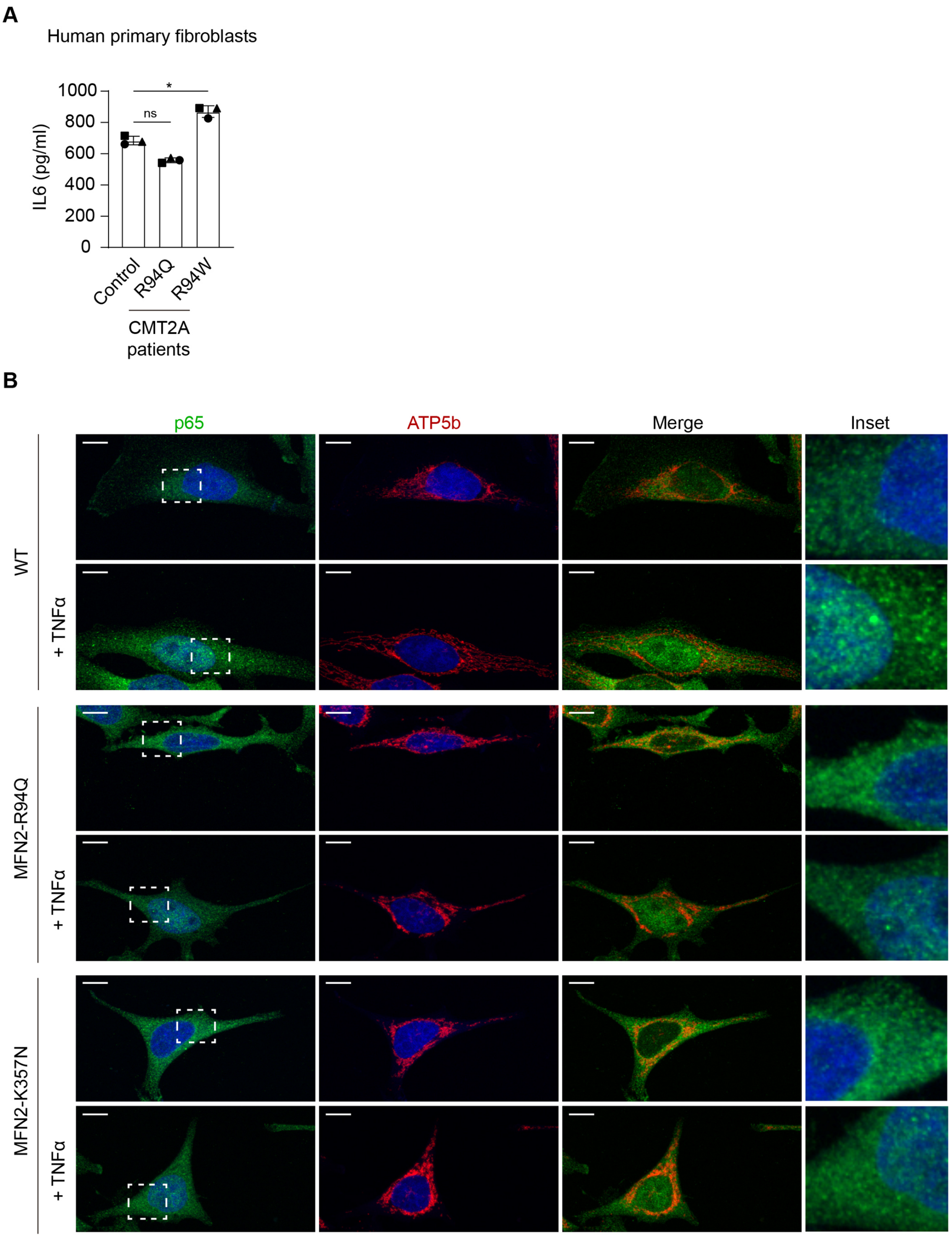

Fig. S7. CMT2A mutant cells do not present visible signs of inflammation. (A) Interleukin 6 concentration was measured in the supernatant of cultures of control or CMT2A patients' primary fibroblasts carrying the point mutations R94Q or R94W in MFN2. **(B)** Confocal images after immunostaining with the inner mitochondrial membrane protein ATP5β (in red), NF-κB subunit p65 (in green) and DAPI (in blue), of HeLa WT and 2KO cells stably expressing MFN2 K357N or R94Q mutant variants of MFN2, untreated or treated with TNF-alpha (25 nM, 15 min).

Journal of Cell Science • Supplementary information

Fig. 1C

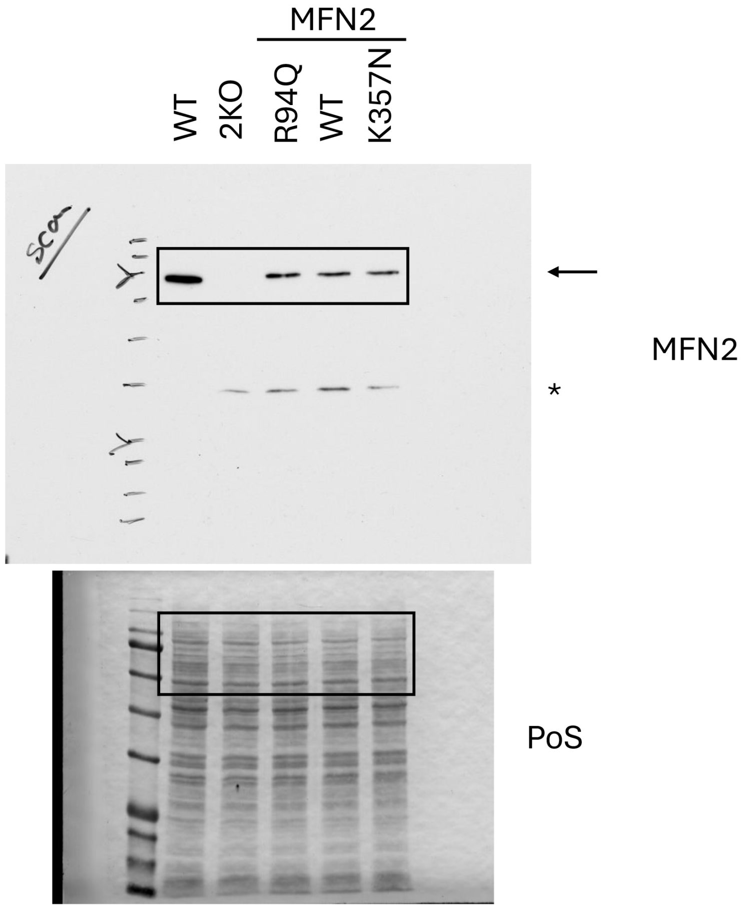

* previous decoration

Fig. 2A

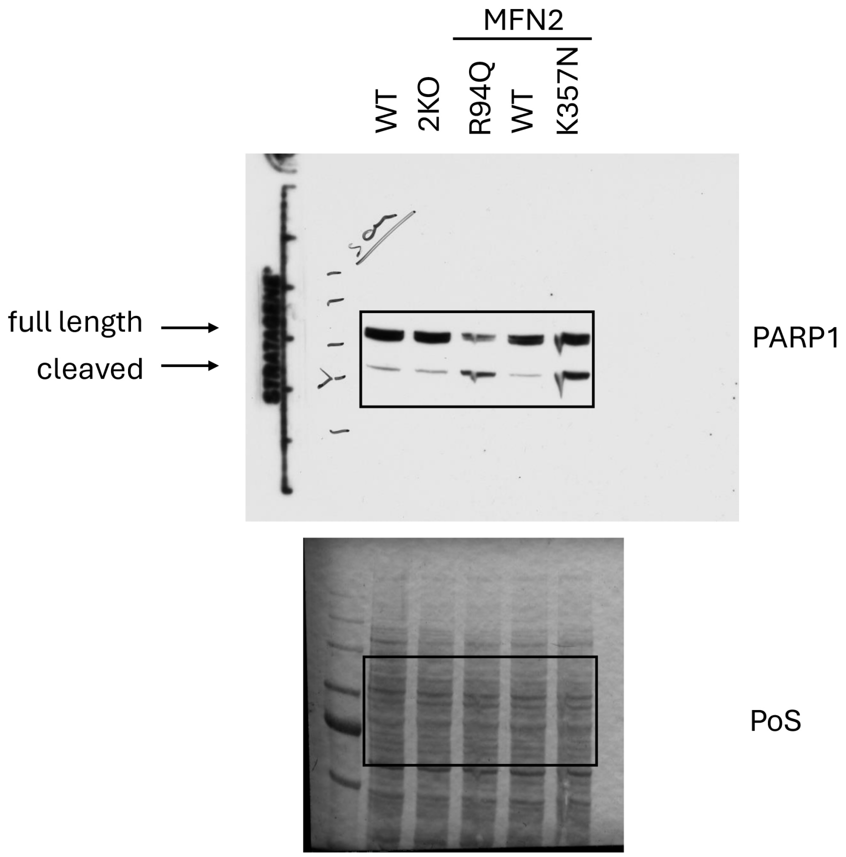

Fig. 2B

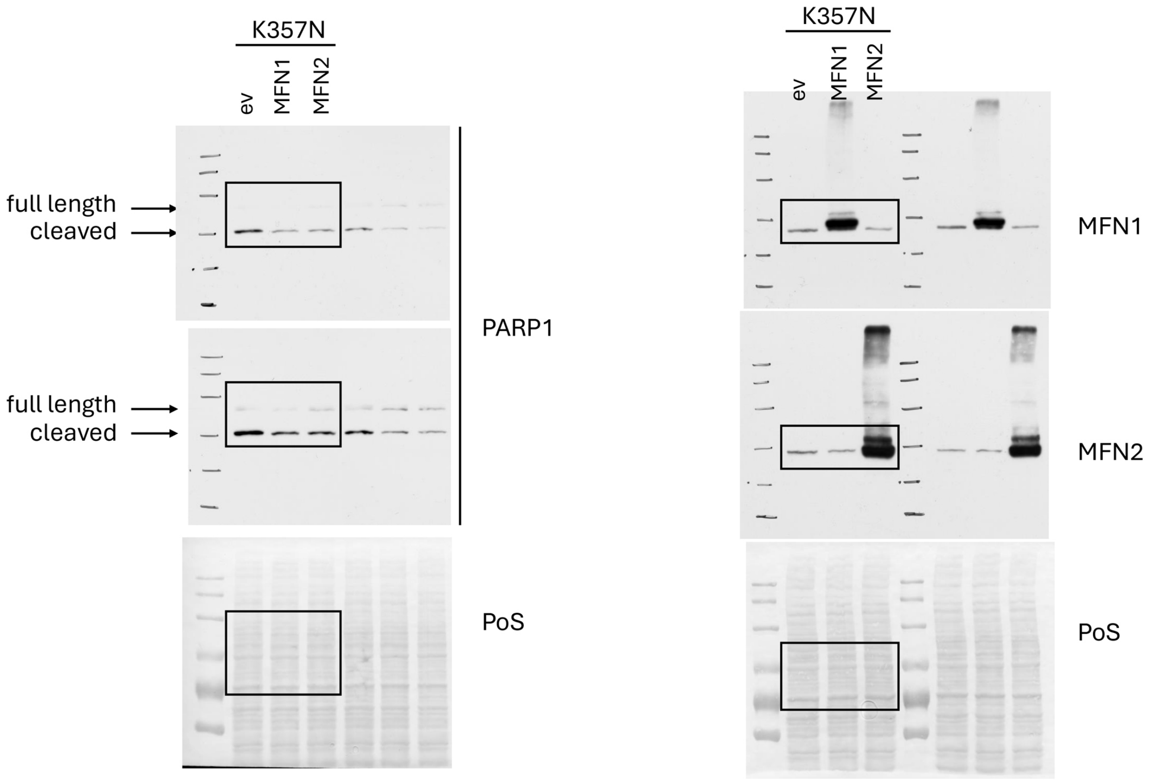

Fig. 2C

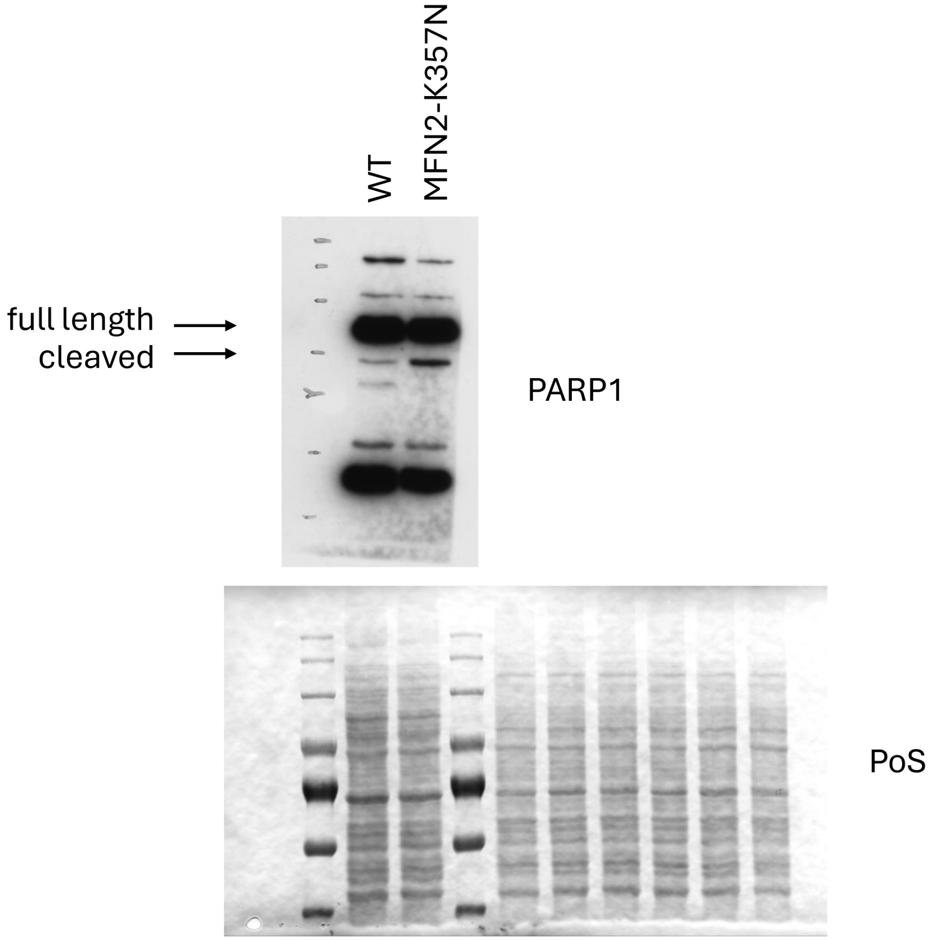

Journal of Cell Science • Supplementary information

Fig. 3A

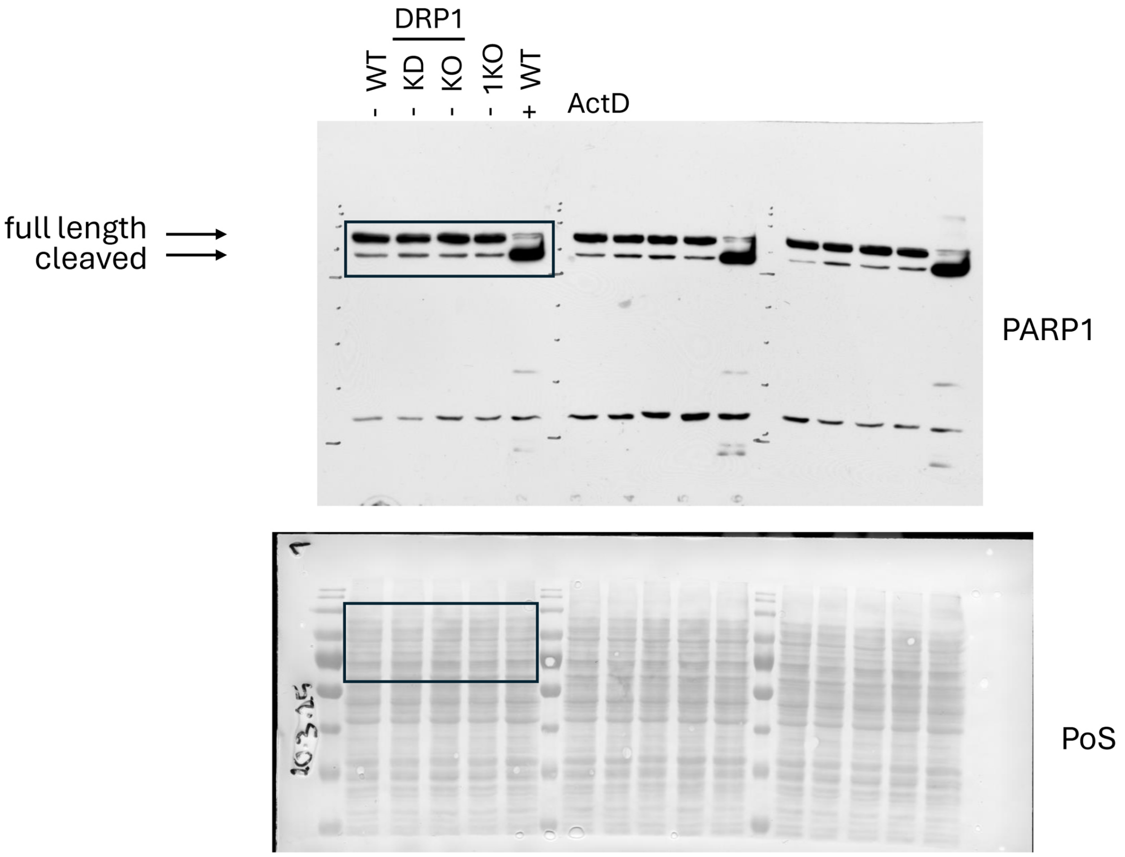

Fig. 3B

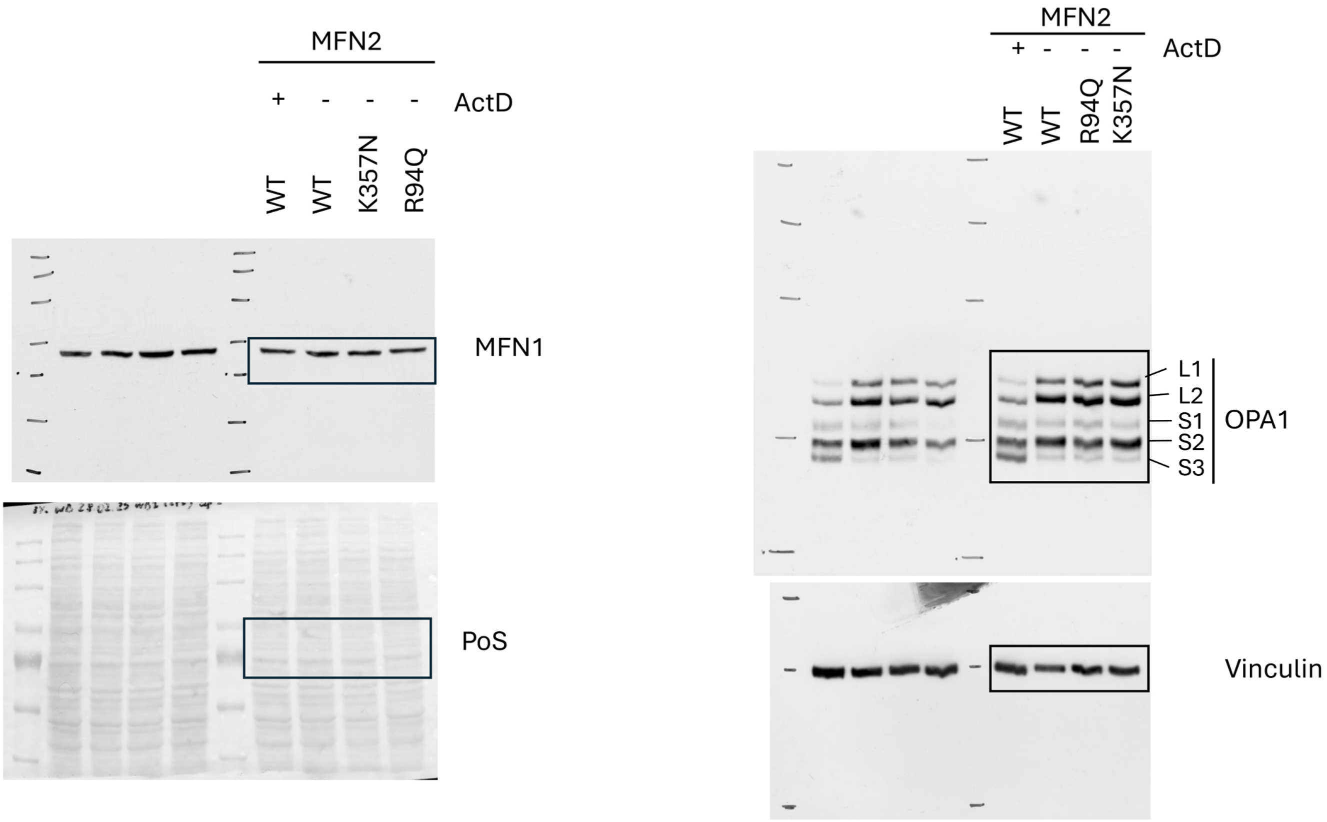

Journal of Cell Science • Supplementary information

Fig. 3C

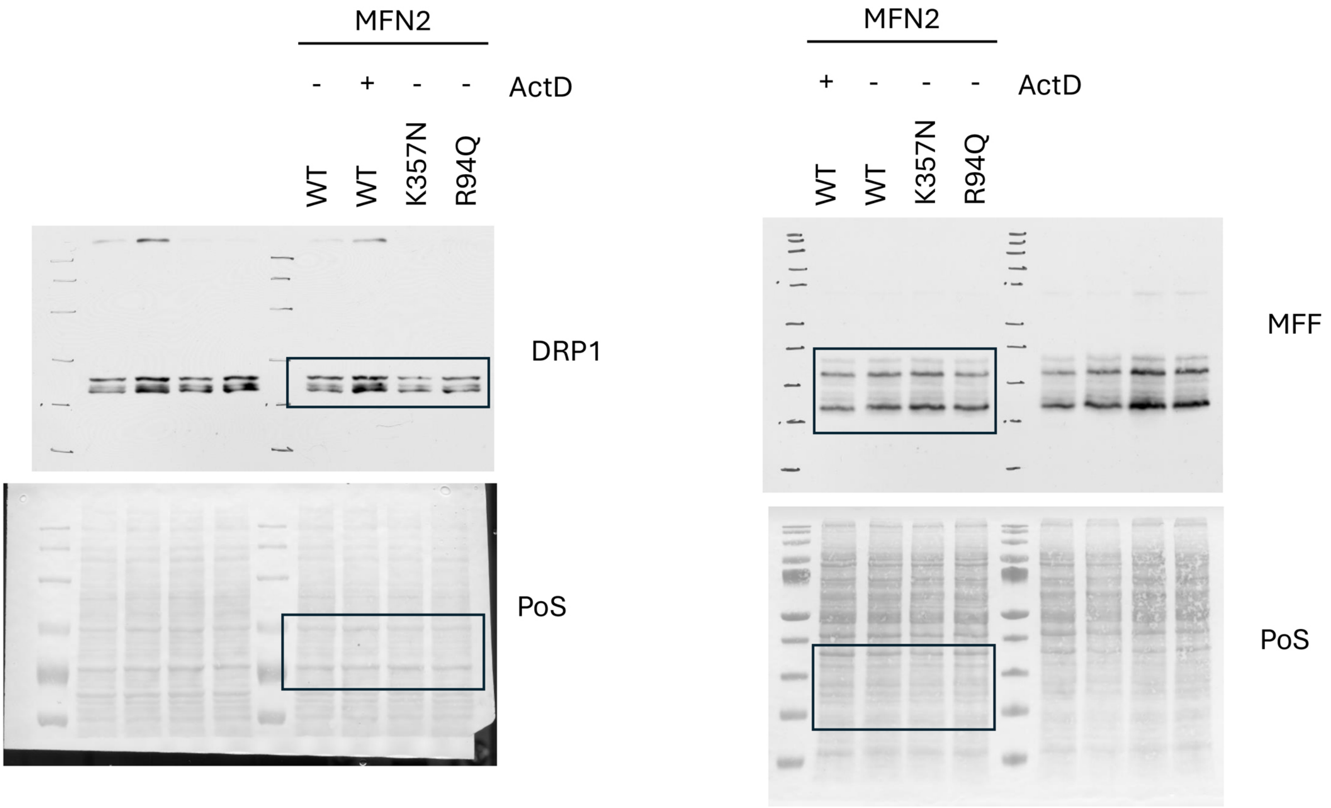

Fig. 4A

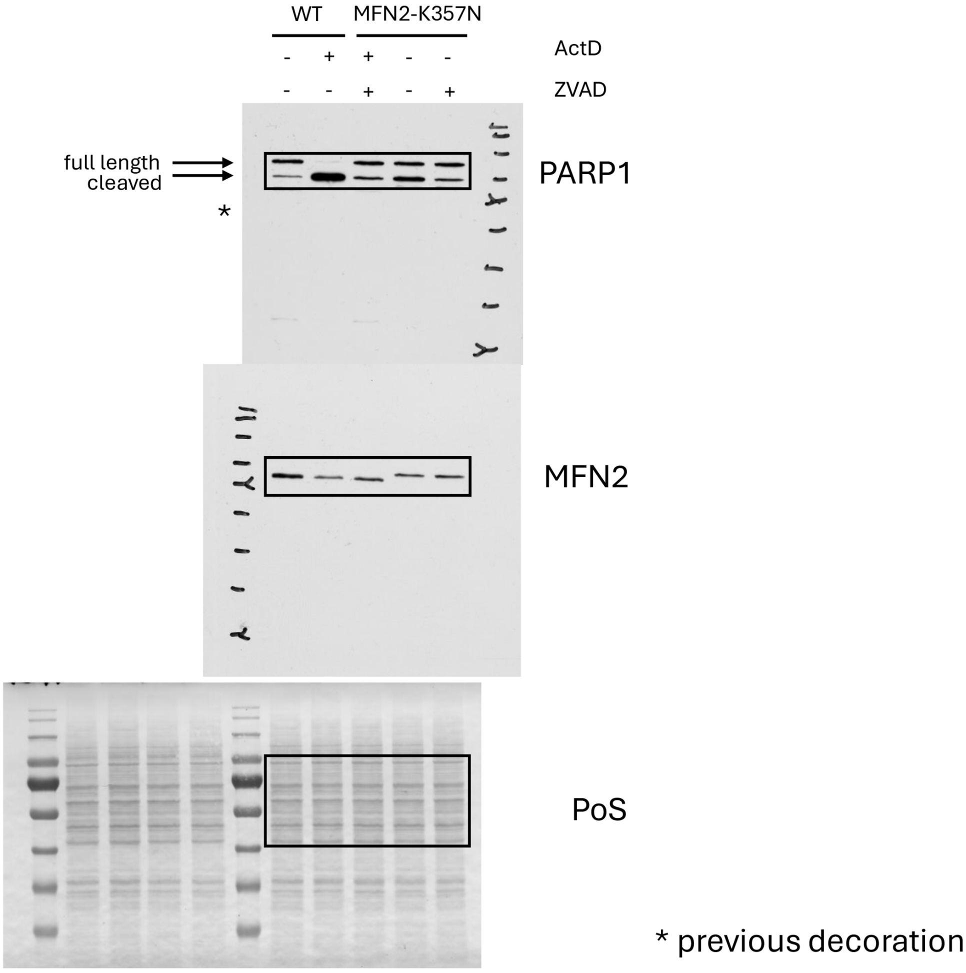

Journal of Cell Science • Supplementary information

Fig. 5A

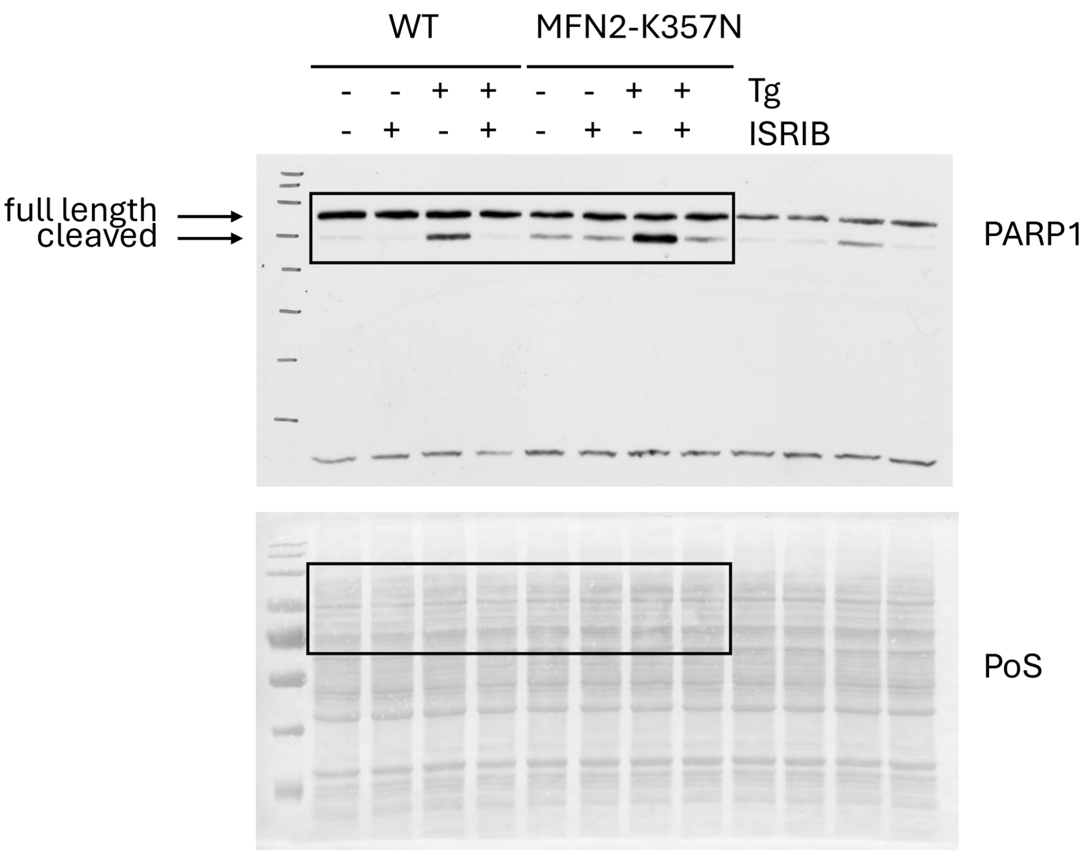
WT
MFN2-K357N
- - + + - - + + Tg
- + - + - + - + ISRIB
full length
cleaved
PARP1
PoS

Fig. 5C

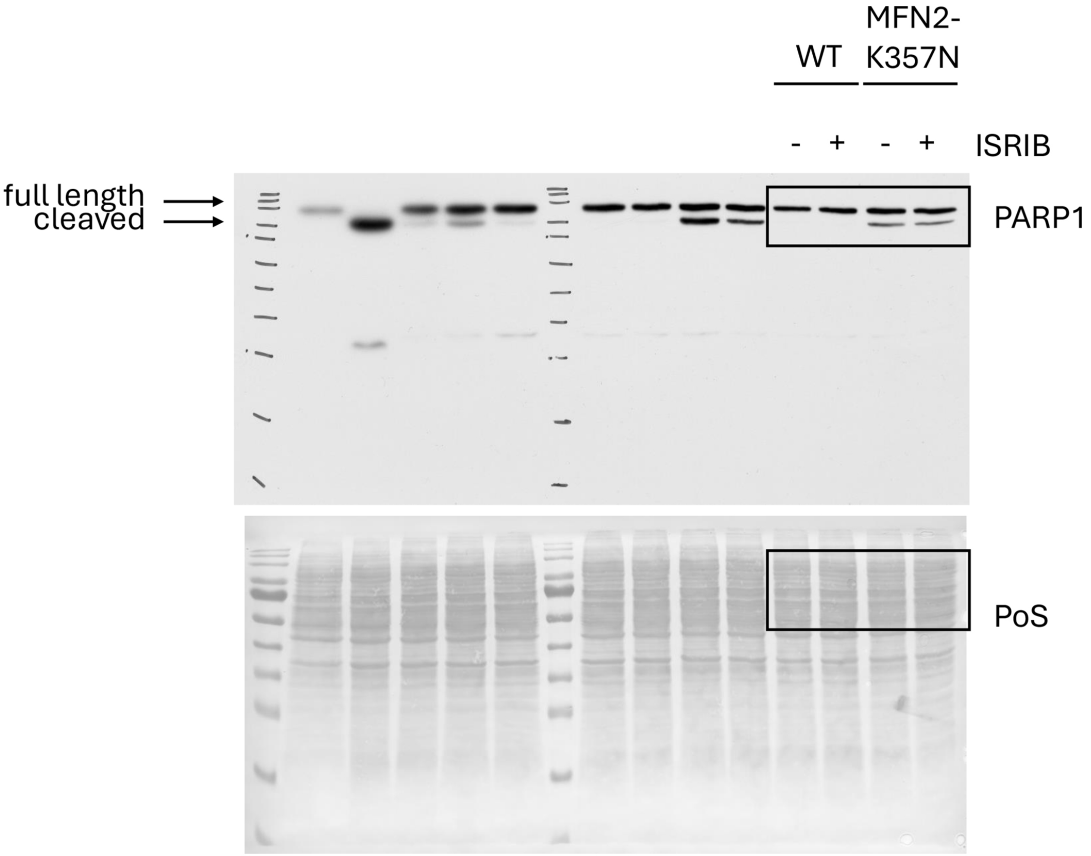
WT
MFN2-K357N
- + - + ISRIB
full length
cleaved
PARP1
PoS

Fig. 6B

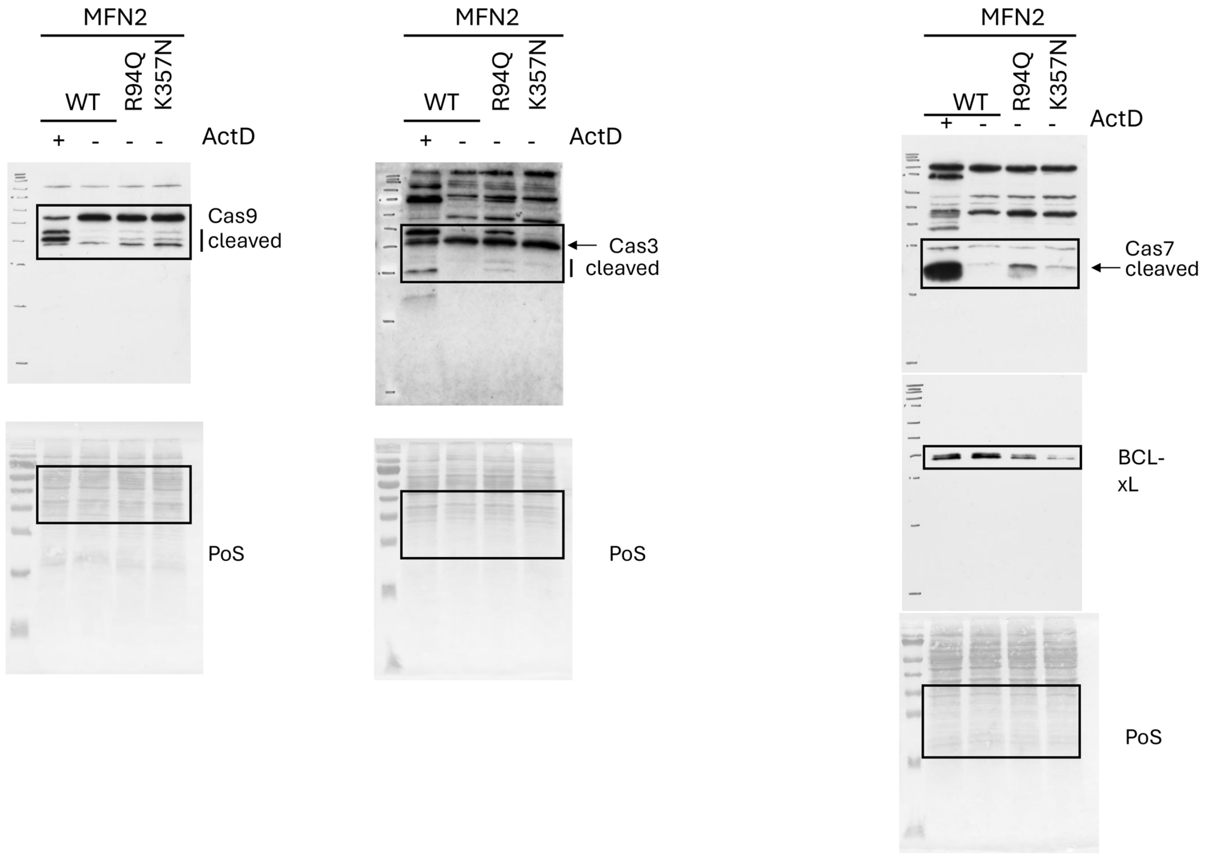

Fig. 6C

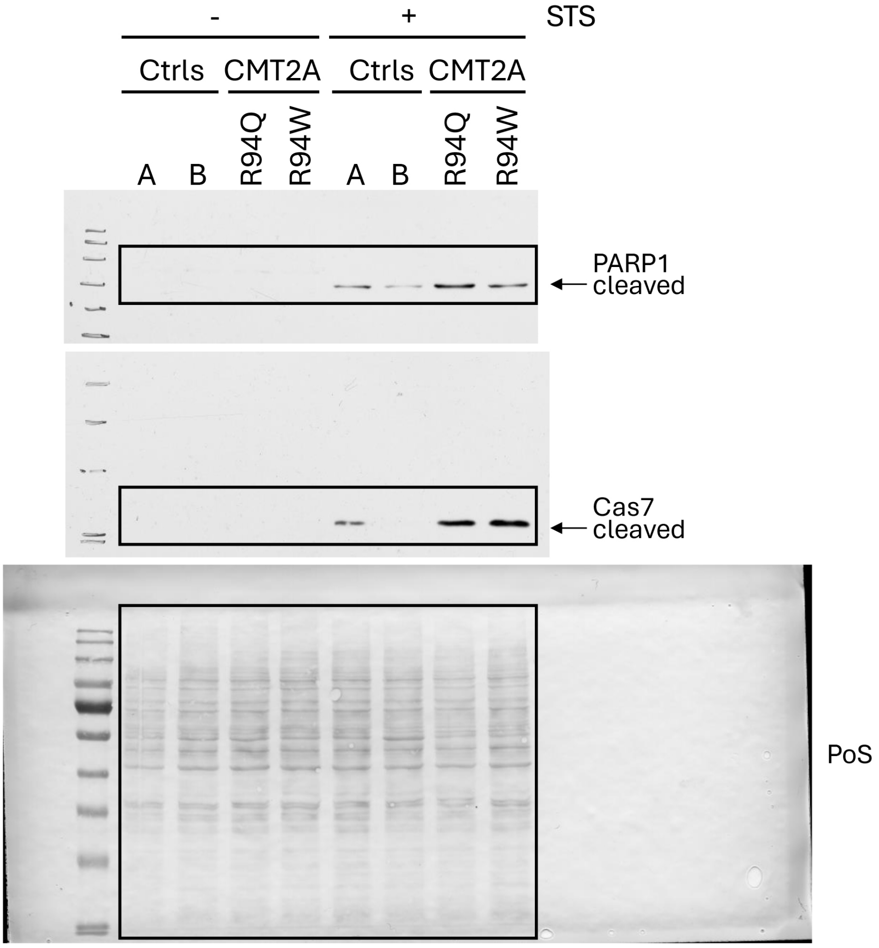

Journal of Cell Science • Supplementary information

Fig. S3A

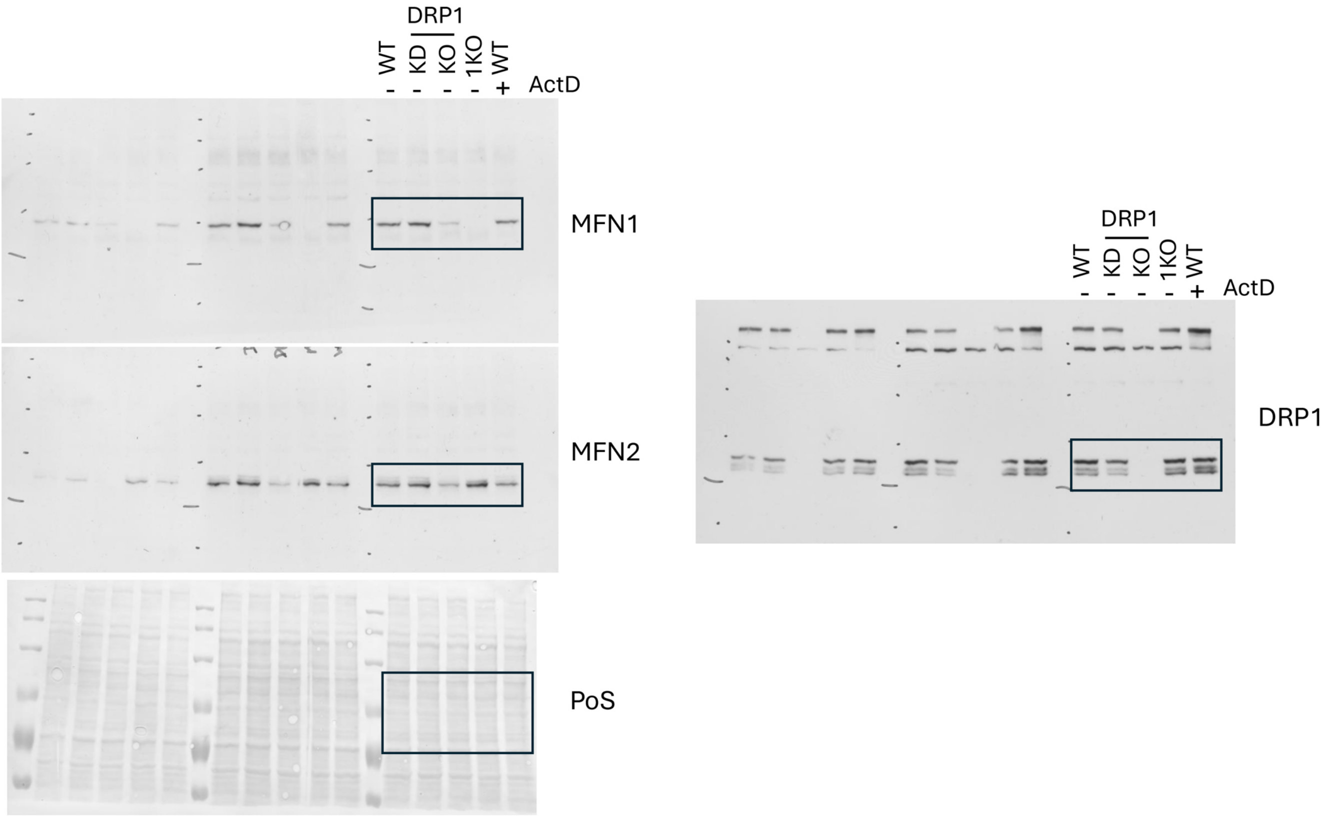

Fig. S3B

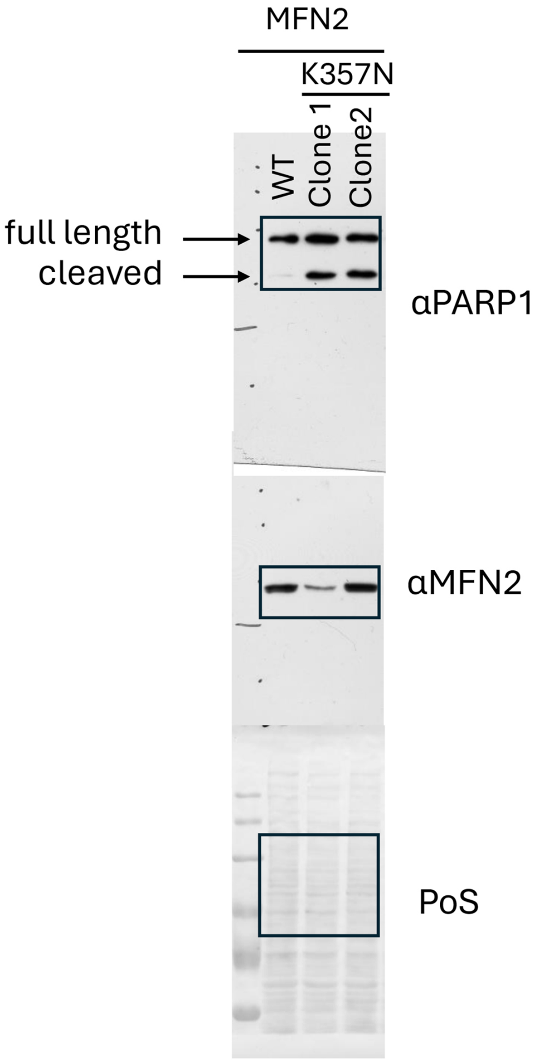

Fig. S3D

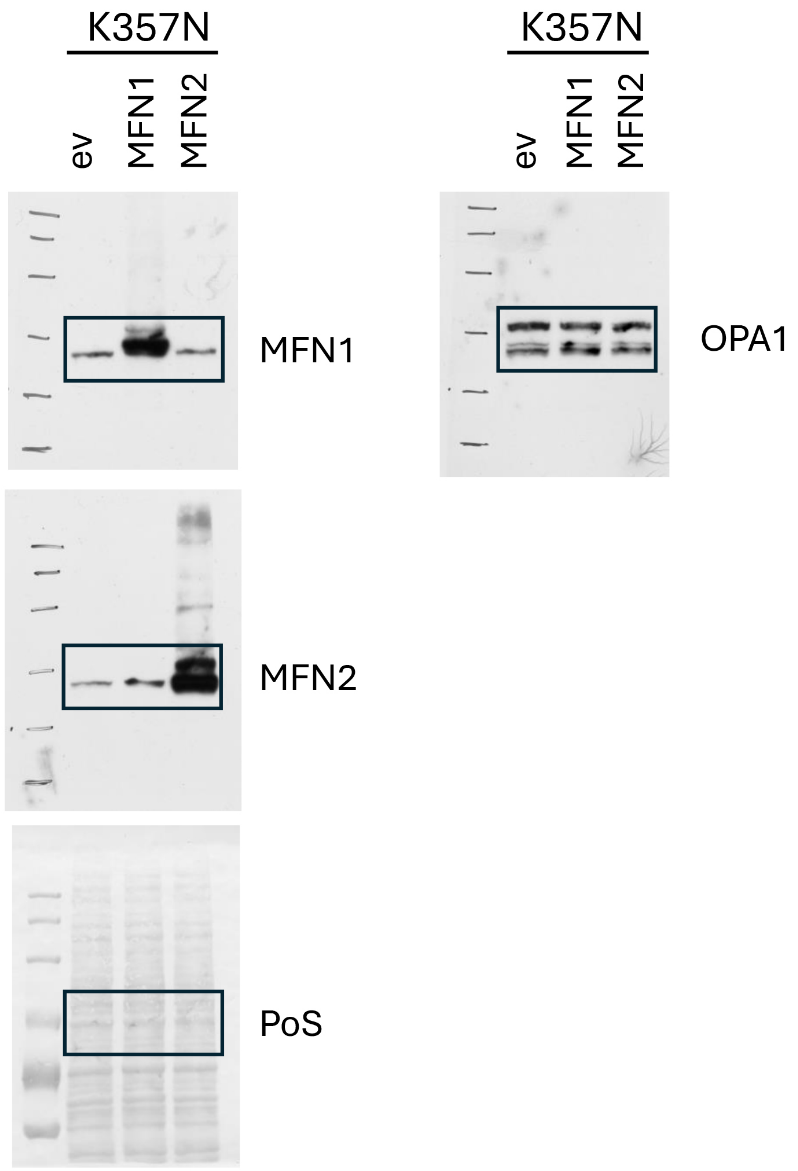

Fig. S8. Blot transparency. Uncropped ponceau (PoS) and film scans of western blot data in the main and supplementary figures.

Journal of Cell Science • Supplementary information

Table S1. Plasmids

Plasmid	Resistance	Reference
PX459 sgRNA DRP1 KO construct 1	Ampicillin	This paper
pCDNA5 (empty vector (ev.)	Ampicillin	Invitrogen
pLVX-puro	Ampicillin	Provided by T. Langer
MFN1-3xFLAG in pCDNA5	Ampicillin	This paper
MFN2-3xFLAG in pCDNA5	Ampicillin	This paper
MFN2-K357N-3xFLAG in pCDNA5	Ampicillin	This paper
MFN2-K109R-3xFLAG in pCDNA5	Ampicillin	This paper
MFN2-R94Q-3xFLAG in pCDNA5	Ampicillin	This paper
MFN2-R707W-3xFLAG in pCDNA5	Ampicillin	This paper
MFN2-P251A-3xFLAG in pCDNA5	Ampicillin	This paper
MFN2-R280H-3xFLAG in pCDNA5	Ampicillin	This paper
MFN2-R364Q-3xFLAG in pCDNA5	Ampicillin	This paper
MFN2-M376L-3xFLAG in pCDNA5	Ampicillin	This paper
MFN2-W740R-3xFLAG in pCDNA5	Ampicillin	This paper
MFN2-R104W -3xFLAG in pCDNA5	Ampicillin	This paper
MFN2-3xFLAG in pLVX-puro	Ampicillin /Puromycin	This paper
MFN2-K357N-3xFLAG in pcDNA5	Ampicillin	This paper
MFN2-R94Q-3xFLAG in pLVX-puro	Ampicillin	This paper

Journal of Cell Science • Supplementary information

Table S2. Antibodies

Antibody	Reference	Dilution	Method
αCHOP	Abcam #ab11419	1:1000	WB
αFLAG	Sigma #F1804	1:1000	WB
αFLAG	Sigma F1804	1:500	IF
αHA	Roche 11867423001	1:1000	WB
αMitofusin 1/2	Abnova H00055669-M04	1:1000	WB
αMitofusin 2	Abcam ab50838	1:1000	WB
αPARP1	Cell Signaling 9542S	1:1000	WB
αTOM20	Sigma HPA011562	1:1000	WB
αTOM20	Sigma HPA011562	1:500	IF
αcleaved Cas3	Cell Signaling 14220	1:1000	WB
αCas3	Cell Signaling 9661	1:1000	WB
αCas7	Cell Signaling 12827S	1:1000	WB
Cleaved αCas7	Cell Signaling 9491S	1:1000	WB
αCas9	Cell Signaling 9502	1:1000	WB
αOPA1	Abcam ab123577	1:1000	WB
αVinculin	Cell Signaling 13901	1:10000	WB
αDRP1	BD Biosciences 611113	1:1000	WB
αMFF	Proteintech 17090-1-AP	1:1000	WB
αFIS1	Santa Cruz sc-376469	1:1000	WB
αBCL-xL	Cell Signaling 2764	1:1000	WB
α-cytochrome c	Life Technologies LT338200	1:200	IF

Journal of Cell Science • Supplementary information

Antibody	Reference	Dilution	Method
αBAX	Cell Signaling 2772S	1:100	IF
αp65	Cell Signaling 4060S	1:500	IF
Goat anti rabbit IgG H&L, CF®568	Biotium, 20102	1:500	IF
Goat anti rabbit IgG H&L, CF®488	Biotium, 20012	1:500	IF
Goat anti mouse IgG H&L, CF®568	Biotium, 20102	1:500	IF
Goat anti mouse IgG H&L, CF®488	Biotium, 20100	1:500	IF
Goat anti-Mouse IgG (H+L), Alexa Fluor™ 633	Invitrogen, A21050	1:500	IF
Goat anti-Rabbit IgG (H+L), Alexa Fluor™ 633	Invitrogen, A21070	1:500	IF
Goat anti-mouse IgG (H+L), HRP-conjugate	Bio-Rad, 1706516	1:5000	WB
Goat anti-rabbit IgG (H+L), HRP-conjugate	Bio-Rad, 1706515	1:5000	WB

Table S3. Primers

Name	Sequence
Guide RNA DRP1_F	CACCGTCGGTGCTCGAGAATTTCGT
Guide RNA DRP1_R	AAACACGAAATTCTCGAGCACCGAC
ATF4_F	GTTCTCCAGCGACAAGGCTA
ATF4_R	ATCCTGCTTGCTGTTGTTGG
ATF6_F	CATCCGCAGAAGGGGAGACA
ATF6_R	CAGGGTCCCACGCTCAGTTT
BiP_F	TGTTCAACCAATTATCAGCAAACTC
BiP_R	TTCTGCTGTATCCTCTTCACCAGT
CHOP_F	AGAACCAGGAAACGGAAACAGA
CHOP_R	TCTCCTTCATGCGCTGCTTT
HPRT_F	GACCAGTCAACAGGGGACAT
HPRT_R	GTGTCAATTATATCTTCCACAATCAAG
XBP1s_F	CTGAGTCCGAATCAGGTGCAG
XBP1s_R	ATCCATGGGGAGATGTTCTGG
